# Characterization of a κ-Carrageenase from Marine *Cellulophaga lytica* strain N5-2 and Analysis of Its Degradation Products

**DOI:** 10.3390/ijms141224592

**Published:** 2013-12-17

**Authors:** Ziang Yao, Feifei Wang, Zheng Gao, Liming Jin, Haige Wu

**Affiliations:** 1 School of Life Science and Technology, Dalian University, Dalian 116622, China; E-Mails: ziangyao@163.com (Z.Y); wff0115@126.com (F.W.); gaozheng0302@126.com (Z.G.); 2 School of Life Science, Dalian Nationalities University, Dalian 116600, China; E-Mail: jlm@dlnu.edu.cn

**Keywords:** κ-carrageenase, *Cellulophaga lytica* strain N5-2, characterization, degradation product

## Abstract

A carrageenan-degrading marine *Cellulophaga lytica* strain N5-2 was isolated from the sediment of carrageenan production base. A κ-carrageenase (EC 3.2.1.83) with high activity was purified to electrophoretic homogeneity from the culture supernatant by a procedure of ammonium sulfate precipitation, dialyzing and gel filtration on SephadexG-200 and SephadexG-75. The purified enzyme was verified as a single protein on SDS-PAGE, and whose molecular weight was 40.8 kDa. The κ-carrageenase yielded a high activity of 1170 U/mg protein. For κ-carrageenase activity, the optimum temperature and pH were 35 °C and pH 7.0, respectively. The enzyme was stable at 40 °C for at least 2.5 h. The enzyme against κ-carrageenan gave a *K_m_* value of 1.647 mg/mL and a *V_max_* value of 8.7 μmol/min/mg when the reaction was carried out at 35 °C and pH 7.0. The degradation products of the κ-carrageenase were analyzed by thin layer chromatography (TLC), high performance liquid chromatography (HPLC), electrospray ionization time-of-flight mass spectroscopy (ESI-TOF-MS) and ^13^C-NMR spectroscopy, and the results indicated that the enzyme was specific of the β-1,4 linkage and hydrolyzed κ-carrageenan into κ-neocarraoctaose-sulfate and κ-neocarrahexaose-sulfate first, and then broke κ-neocarraoctaose-sulfate into κ-neocarrabiose-sulfate and κ-neocarrahexaose-sulfate.

## 1. Introduction

Carrageenans are linear sulfated galactans extracted from many species of red seaweeds and share a common backbone of d-galactose with alternating α-1,3 and β-1,4 linkages. They are classified according to the number and the position of sulfate ester groups. The main industrially exploited carrageenans are κ- and ι-carrageenans owing to their gelling properties. κ-carrageenan is conventionally described as the repetition of the disaccharide motif 4-sulfate-*O*-β-d-galactopyranosyl-(1,4)-3,6-anhydro-α-d-galactose. In most reports, κ-carrageenase, which hydrolyzes β-1,4 linkages in κ-carrageenan, is a novel family-16 glycoside hydrolases member [1,2]. κ-carrageenase as a tool enzyme for carrageenan degradation has a wide range of applications in the formation of algal protoplast [3], preparation of carrageenan oligosaccharides [4], structure and function study of carrageenan [5,6], *etc*. Furthermore, compared with carrageenan, carrageenan oligosaccharide and its derivants have more biological activities including anti-tumor [7], anti-oxidation [8], anti-cogulant, immunoregulation and anti-angiogenesis [9]. Among the previous reports, κ-carrageenases have been isolated from Pseudoalteromonas [10], Cytophaga [11], Alteromonas carrageenovora and Pseudomonas carrageenovora [12,13]. In the present study, a κ-carrageenase was purified from *Cellulophaga lytica* strain N5-2, and the hydrolyzed products of the enzyme were analyzed.

## 2. Results and Discussion

### 2.1. Identification of Marine *Cellulophaga lytica* Strain N5-2

**Figure 1.**
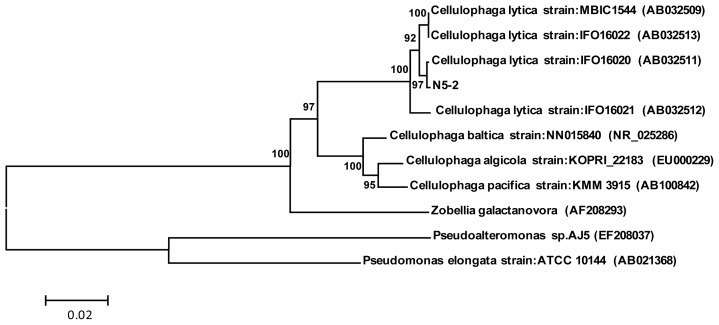
Neighbor-joining phylogenetic tree based on 16SrRNA gene sequencing showed the relationships between the strain of N5-2 and other related genera. Neighbor-joining phylogenetic tree based on 16Sr RNA gene sequencing showed the relationships between the strain N5-2 and other related genera. Numbers at nodes are levels of bootstrap support (%). Scale bar represents two nucleotides substitution per 100 nucleotides.

Marine *Cellulophaga lytica* strain N5-2 produced yellow pigmentation and displayed gram negative, the cell size was 0.6 μm × (2.5–3.7) μm; colonies were yellow with irregular edge and granular surface protuberance. The 16SrRNA sequence of this strain demonstrated 99% similarity with the *Cellulophaga lytica* strain IFO: 16020 (AB032511). Therefore, the phylogenetic tree suggested that the strain may be a new strain in the *Cellulophaga lytica* (Figure 1), called *Cellulophaga lytica* strain: N5-2. The nucleotide sequence of 16S rRNA of the strain was submitted to GenBank nucleotide sequence database. The sequence is available in the GenBank nucleotide sequence database with the accession number of GU129978.

### 2.2. Purification of κ-Carrageenase

**Figure 2.**
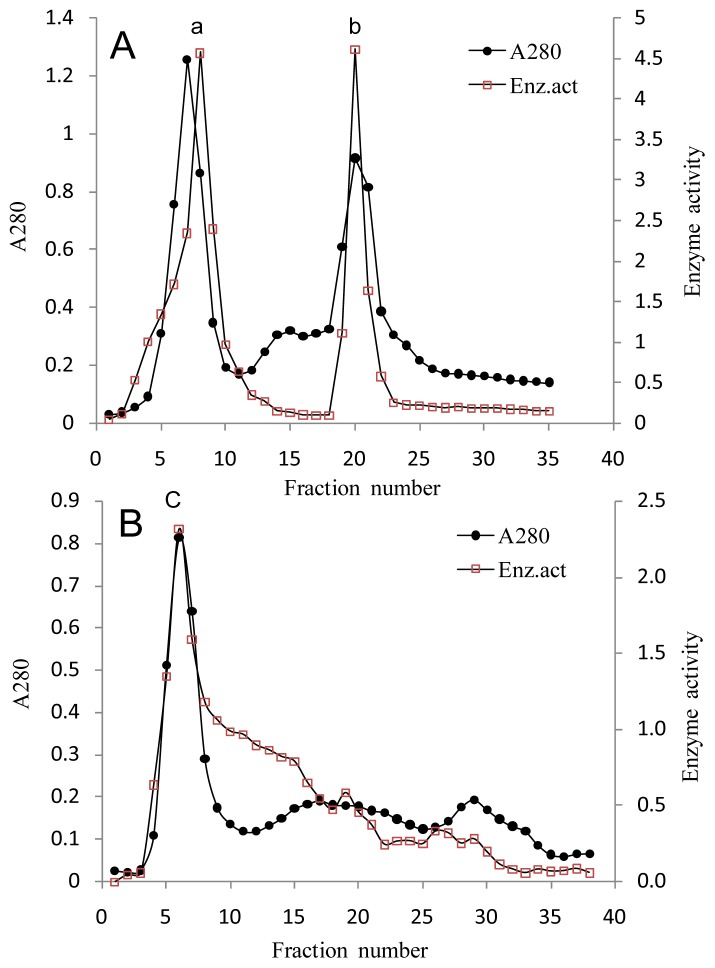
Gel filtration chromatography of κ-carrageenase. (**A**) Sephadex G-200 gel filtration chromatography; and (**B**) Sephadex G-75 gel filtration chromatography. The sample was eluted at a flow rate of 0.1 mL/min with Tris-HCl buffer at pH 7.0. The eluates were monitored for protein (A280) and κ-carrageenase activity.

**Figure 3.**
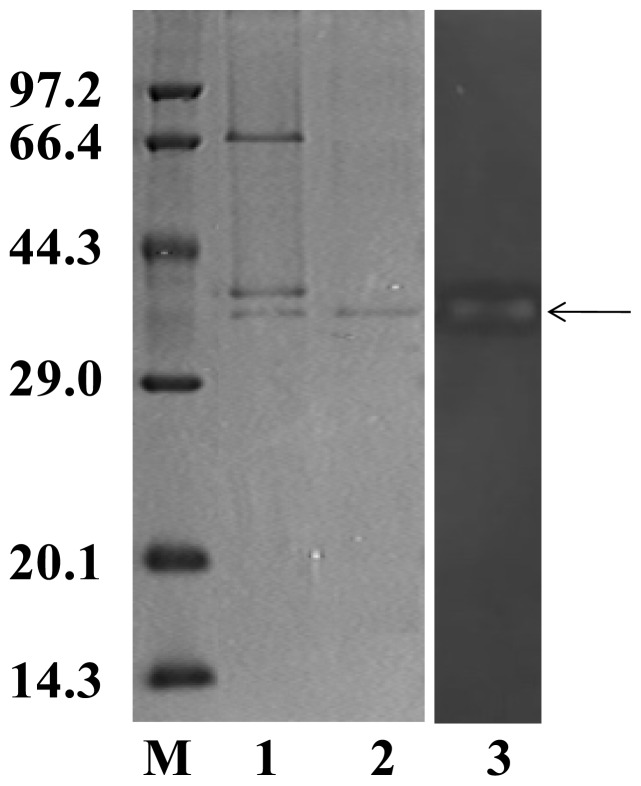
SDS-PAGE pattern and zymography of purified κ-carrageenase. SDS-PAGR was conducted in a 5%/15% discontinuous polyacrylamide gel at 25 mA at room temperature and stained with Coomassie brilliant blue R-250. The peaks from Sephadex G-200 contained three protein bands (**1**); Only one protein band, with an apparent molecular mass of 40.8 kDa, was detected in the activity peak from Sephadex G-75 (**2**); The sample of the activity peak from Sephadex G-75 was applied to zymography experiment, stained with alcian blue. A degradated band was detected at the same position, just as pointed by the arrow (**3**). Line **M** was protein molecular mass marker.

As shown in Table 1, after precipitated with 40% and 80% (NH_4_)_2_SO_4_, about 80% foreign protein was removed and the specific activity was increased about 4 folds with a significant loss of total activity (69.5% recovery). Sephadex G-200 column chromatography separated 2 peaks with κ-carrageenase activity (Figure 2A), by this step the specific activity was increased 23 folds. The further purification of κ-Carrageenase was obtained by Sephadex G-75 column chromatography, from which a single activity peak (Figure 2B) was achieved. The result of SDS-PAGE showed that this activity peak contained a single protein band (Figure 3 line 2) with molecular mass of 40.8 kDa. A clear zone on zymography further stained the band could degrade κ-carrageenan (Figure 3 line 3). The final purified enzyme yielded significantly high activity of 1170 U/mg protein and a fold of 40 (Table 1), at the same time much of the total activity was lost with 17.3% recovery only.

### 2.3. Properties of the κ-Carragcenase

**Figure 4.**
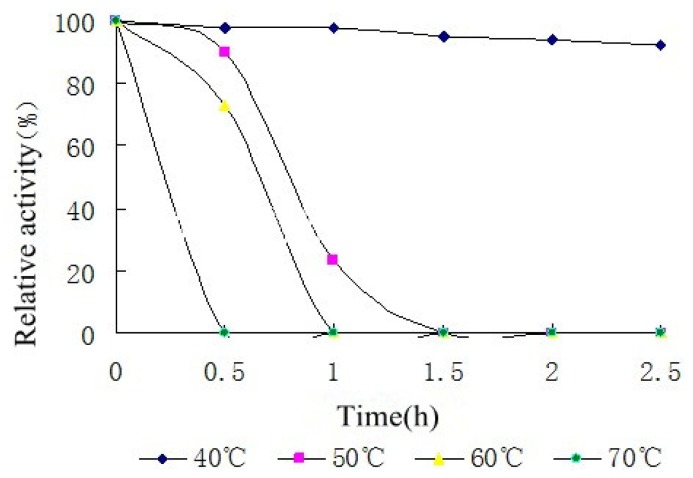
Temperature stability of the κ-carrageenase from *Cellulophaga lytica* strain N5-2. The enzyme solution was incubated at each temperature (20–70 °C) for 0.5 to 2.5 h and then the residual enzyme activity was measured. The activity of untreated enzyme was regarded as 100% and relative activity was determined.

κ-carrageenase was stable in a broad range of temperature (20 °C to 60 °C) and 35 °C was optimum temperature. Furthermore, temperature stability result showed that the κ-carrageenase had a good thermal stability at 40 °C, with the prolongation of time the enzyme activity remained stable at least for 2.5 h. When the temperature was above 40 °C, the enzyme activity decreased significantly as time prolonging (Figure 4).

**Figure 5.**
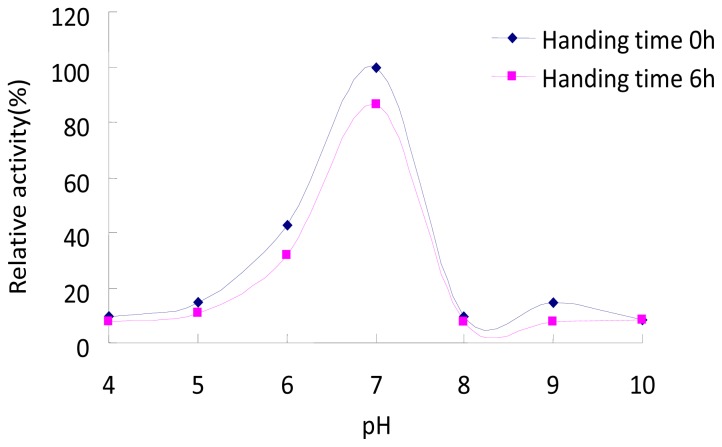
pH stability of the κ-carrageenase from *Cellulophaga lytica* strain N5-2. The different pH buffer was: Na_2_HPO_4_-Citric acid buffer (pH 4.0–7.0), Tris-HCl buffer (pH 8.0 and 9.0) and glycine-NaOH buffer (pH 10.0). Pre-incubating the enzyme solution at each pH (4.0–10.0) at 35 °C for 6 h and then the enzyme activity was determined in the same pH buffer. The activity of untreated enzyme was regarded as 100% and relative activity was determined.

The optimum pH of the κ-carrageenase was 7.0. The enzyme was inactivated or denaturated at extreme values of pH, and handling time had a weak effect on the enzyme activity (Figure 5).

As calculated from Lineweaver plots, the aparent *K_m_* and *V_max_* values of the κ-carrageenase were 1.647 mg/mL and 8.7 μmol/min/mg respectively.

### 2.4. Analysis of Degradation Products

**Figure 6.**
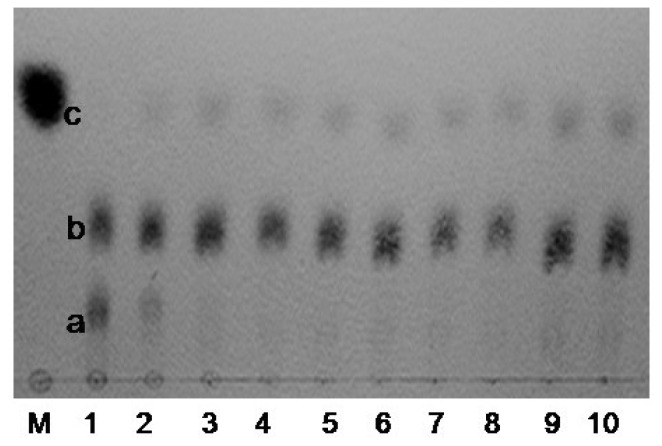
Thin layer chromatography (TLC) of the κ-carrageenase degradation products. Enzymatic hydrolysis of κ-carrageenan was conducted under standard condition with 0.5% κ-carrageenan as substrate and reaction for 5 min (**1**); 30 min (**2**); 1 h (**3**); 2 h (**4**); 3 h (**5**); 6 h (**6**); 9 h (**7**); 12 h (**8**); 24 h (**9**) and 48 h (**10**), respectively. **M**, control (galactose). Combined with HPLC and mass spectrometry results, a, b and c were ê-neocarraoctaose, κ-neocarrabiose and κ-neocarrabiose respectively.

**Figure 7.**
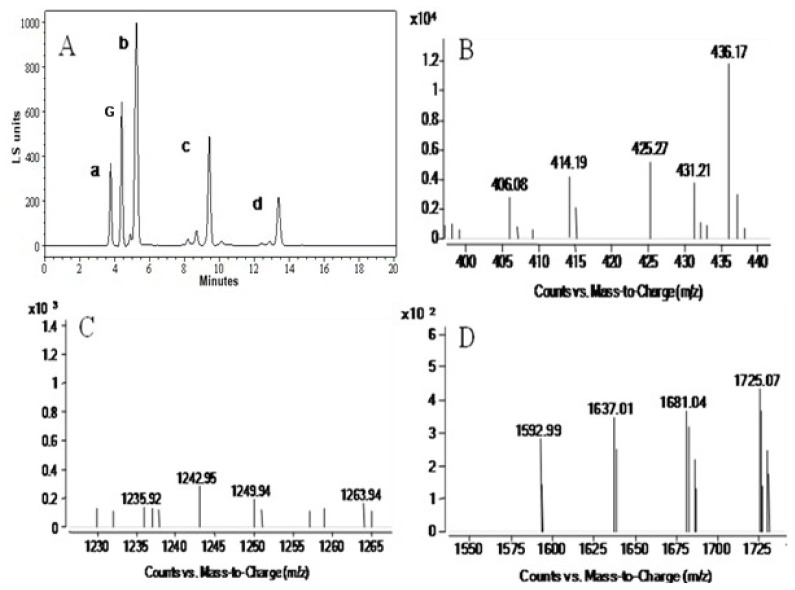
HPLC (**A**) and MSI-TOF-MS (**B**–**D**) analysis of the κ-carrageenase degradation products. (**A**) HPLC spectrum of the κ-carrageenase degradation products. κ-carrageenan was hydrolyzed with the enzyme under standard condition for 48 h, and the product was separated with HPLC. Three components were detected and separated (b, c and d), a is solvent peak, G is control (galactose) peak. The components obtained from HPLC were analyzed with MSI-TOF-MS to determine the molecular mass distribution; (**B**–**D**) MSI-TOF-MS analysis of the κ-carrageenase degradation products b, c and d (in HPLC) respectively.

Analysis of the time course of the oligosaccharide release showed that at first κ-carrageenan was hydrolyzed into two high-molecular-mass components a and b (Figure 6), with the prolongation of hydrolysis time, a smaller molecular-mass component c emerged and component-a decreased gradually. The hydrolyzed product contained three components a, b and c were separated with HPLC, three dominant peaks appeared on HPLC mass spectrum (Figure 7A–D).

In order to identify the molecular mass of the hydrolyzed products, the three components obtained from HPLC was analyzed with MSI-TOF-MS, and the result demonstrated that the molecular mass of three components were 425.27, 1242.95 and 1681.04 Da respectively (Figure 7B–D). It is well known that the base unit of the κ-carrageenan was a dimer (426 Da) composed of one β-d-galactopyranose residue (G-unit) and one 3, 6-anhydro-α-d-galactopyranose residue (A-unit). So the 425.27 Da component could be κ-neocarrabiose-sulfate, the 1242.95 Da component could be κ-neocarrahexaose-sulfate, and the molecular mass of acetylated κ-neocarraoctaose was 1681.04 Da.

**Figure 8.**
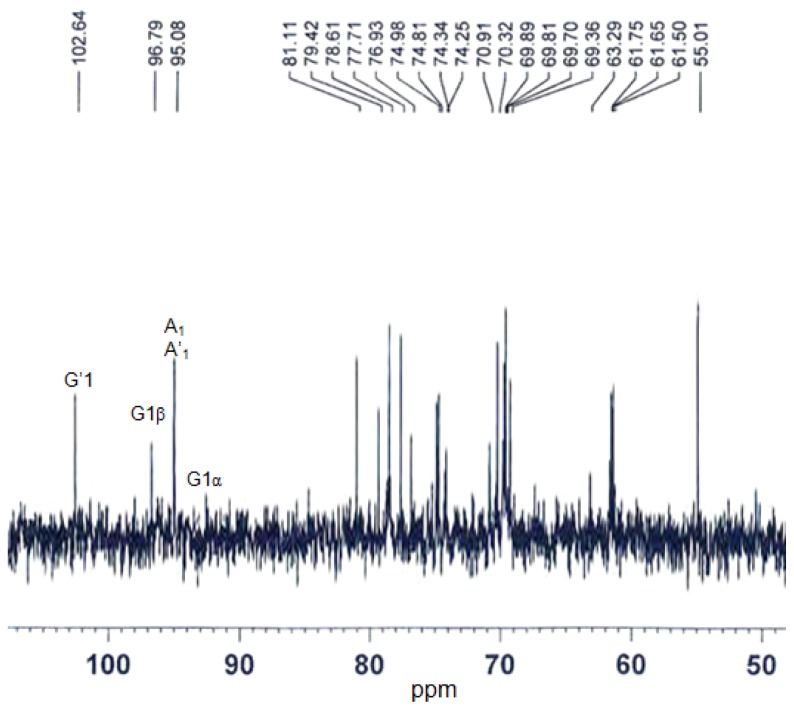
^13^C-NMR spectrum of the κ-carrageenase degradation products. The degradation products were dissolved in ^2^H_2_O and processed at 30 °C. Spectrum was recorded on an AVANCE 500 MHz apparatus (Bruker, Karlsruhe, Germany).

The structure information of the products was further obtained by ^13^C-NMR. A typical pattern for oligosaccharide was observed according to the NMR spectrum (Figure 8). Only one signal was observed at around 102.64 ppm, which is the characteristic signal of the G-unit at non-reducing end of the oligosaccharides. The resonance at 95.08 ppm was assigned to be the carbon of the A-unit. The signal at 92.5 and 96.79 ppm were assigned to be the carbon of the G-unit at the reducing end for α and β configuration respectively. According to above results, the reducing end of the oligosaccharides was G-unit. This indicated that the κ-carrageenase from N5-2 is specific of the β-1,4 linkage. So the hydrolyzed products of the κ-carrageenase were κ-neocarrabiose-sulfate, κ-neocarrahexaose-sulfate and κ-neocarraoctaose-sulfate respectively.

**Figure 9.**
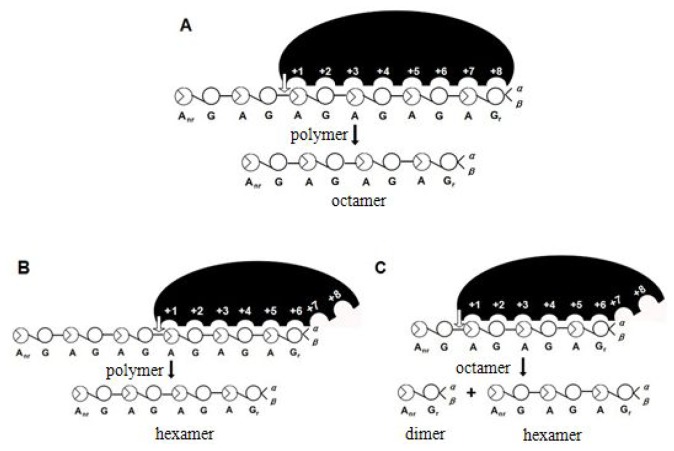
Degradation mode of κ-carrageenase. The recognition units of the enzyme are κ-neocarraoctaose (**A**) and κ-neocarrahexaose (**B**); and then κ-neocarraoctaose is broken into κ-neocarrabiose and κ-neocarrahexaose (**C**). The end-products of the κ-carrageenase are κ-neocarrabiose and κ-neocarrahexaose.

It is surprising that neocarratetraose-sulfate was not detected suggesting that the recognition units of the enzyme were κ-neocarraoctaose and κ-neocarrahexaose, and then κ-neocarraoctaose was broken into κ-neocarrabiose and κ-neocarrahexaose (Figure 9).

## 3. Materials and Methods

### 3.1. Microorganism

*Cellulophaga lytica* strain N5-2 was isolated from the sediment of carrageenan production base in Hainan (Hainan, China), and cultured according to our previous method [14].

### 3.2. Chemicals

κ-carrageenan was purchased from the Changhang Colloid Technological Co., Ltd. (Changzhou, Jiangsu, China). SephadexG-200 and SephadexG-75 are of Pharmacia products (Pharmacia Company, Stockholm, Sweden). All other reagents were of analytical grade.

### 3.3. κ-Carrageenase Assay

κ-carrageenase activity was determined by measuring the increase in the concentration of reducing sugar as described by Kidby [15]. The assay system consisted of 1.0 mL substrate [0.5% κ-carrageenan in Na_2_HPO_4_-citric acid buffer (pH 7.0)] and 1.0 mL enzyme, and boiled inactivated enzyme with the same treatment was used as control. One unit of κ-carrageenase activity was defined as the amount of enzyme needed to release 1 μmol reducing sugars (d-galactose equivalent) per min.

### 3.4. Purification of κ-Carrageenase

Strain N5-2 was propagated in a medium containing: κ-carrageenan (2 g), FeSO_4_·7H_2_O (0.02 g), polypepton (3 g) in 1000 mL layout seawater with shaking for 20 h at 35 °C. The following operations were carried out at 4 °C. The culture medium was centrifuged (10,000 *g*, 20 min) and the cell-free supernatant was fractionated at 40% and 80% ammonium sulfate saturation. The precipitated protein with 40% ammonium sulfate saturation was discarded, and the precipitated protein with 80% ammonium sulfate saturation was resuspended in distilled water and dialyzed in dialysis bag (MWCO: 8000–14,000 Da) against the distilled water, and freeze-dried successively. Protein contents were determined by Bradford method [16]. The obtained enzyme powder was dissolved in 5 mL Tris-HCl buffer (pH 7.0) and the end concentration was 4%, then the enzyme solution was applied to Sephadex G-200 (Pharmacia company, Stockholm, Sweden) column chromatography (100 × 1.6 cm) equilibrated with the same buffer, and eluted at a flow rate of 0.1 mL/min. The eluates were monitored continuously at 280 nm for protein and fractions were assay for activity against κ-carrageenan. All the fractions of the first peak from Sephadex G-200 column chromatography containing κ-carrageenase activity were gathered, concentrated and applied to another column of Sephadex G-75, furthermore equilibrated with the same eluent. Fractions were collected and monitored for the presence of κ-carrageenase. The purity of the fractions was assessed by SDS-PAGE. Pure fractions with activity were stored at −20 °C.

### 3.5. Zymography Experiment

15% Separating gel was prepared by adding 0.2% κ-carrageenan, the content of other reagents was as same as the normal SDS-PAGE. The zymography electrophoresis was carried out as normal discontinuous SDS-PAGE. After electrophoresis, above all, the separating gel was renaturated in renaturation solution (0.05 mol/L Tris–HCl, 0.2 mol/L NaCl, 0.01 mol/L CaCl_2_, 1% TritonX-100, pH 7.0) at 35 °C for 5 h, then stained with 0.1% alcian blue (in 0.1 M HCl, pH 1.0).

### 3.6. Dependence and Stability of the Enzyme Activity on Temperature and pH

The optimum temperature for κ-carrageenase activity was determined under the standard assay condition by varying the incubation temperature from 20 to 70 °C. The thermal stability of the κ-carrageenase was determined by incubating the enzyme solution at each temperature (20–70 °C) for 0.5 to 2.5 h and then measuring the residual enzyme activity. The activity of untreated enzyme was regarded as 100% and relative activity was determined.

The effect of pH on κ-carrageenase activity was assayed by replacing Na_2_HPO_4_-citric acid buffer (pH 7.0) with: Na_2_HPO_4_-citric acid buffer (pH 4.0–7.0), Tris-HCl buffer (pH 8.0 and 9.0) and glycine-NaOH buffer (pH 10.0) at 35 °C. The pH stability of the κ-carrageenase was determined by pre-incubating the enzyme solution at each pH (4.0–10.0) at 35 °C for 6 h and then the enzyme activity was determined in the same pH buffer. The activity of untreated enzyme was regarded as 100% and relative activity was determined.

### 3.7. Kinetic Studies

The initial reaction rate of κ-carrageenan degradation were assayed at several κ-carrageenan concentrations (0.3125, 0.625, 1.25, 2.5 and 5.0 mg/mL) by DNS method and the reaction system contained 0.5 mg/L pure enzyme protein. The Michaelis constant (*K*_m_) and the reaction rate at infinite substrate concentration (*V*_max_) were determined according to the Lineweaver-Burk plotting method [17].

### 3.8. Analysis of Degradation Products

Enzymatic hydrolysis of κ-carrageenan was conducted under standard condition with 0.5% κ-carrageenan as substrate and reaction for 5 min, 30 min, 1 h, 2 h, 3 h, 6 h, 9 h, 12 h, 24 h and 48 h, respectively. The products of different reaction time was analyzed by TLC (thin-layer chromatograthy) method with the developing agent composed of butanol–acetic-acid–water (2:1:1, *v*/*v/v*) [3], and the saccharide was visualized with a diphenylamine-aniline-phosphate reagent.

κ-carrageenan was hydrolyzed with the enzyme under standard condition for 48 h, and the product was separated with HPLC (Agilent 1100, Agilent Technologies, Santa Clara, CA, USA), and the separation condition was moving phase 2.5 mM ammonium acetate:methyl alcohol (3:2). The components obtained from HPLC were analyzed with ESI-TOF-MS (Agilent G1969A, Agilent Technologies, Santa Clara, CA, USA) to determine the molecular mass distribution. Structure of the product was identified by ^13^C-NMR spectroscopy (Bruker AVANCE 500 MHz, Bruker, Karlsruhe, Germany), 10 mg sample was dissolved in 0.5 mL H_2_O and assay was carried out at 30 °C.

## 4. Conclusions

In this paper, a marine bacterium with high κ-carrageenase activity was isolated and was identified as marine *Cellulophaga lytica* by 16SrRNA, named strain N5-2. After purification, the specific activity of the enzyme was 1170 U/mg. The molecular mass of the enzyme was 40.8 kDa, and it possessed thermal, pH stabilities and satisfactory kinetic property. This κ-carrageenase from N5-2 was specific of the β-1,4 linkage, and the recognition units of the enzyme were κ-neocarraoctaose and κ-neocarrahexaose, and then κ-neocarraoctaose was broken into κ-neocarrabiose and κ-neocarrahexaose. So the hydrolyzed products of the enzyme were κ-neocarrabiose-sulfate, κ-neocarrahexaose-sulfate and κ-neocarraoctaose-sulfate respectively.

## Figures and Tables

**Table 1.** Purification of the κ-carrageenase from *Cellulophaga lytica* strain N5-2.

Step	Volume (mL)	Total protein a (mg)	Total activity b (units)	Specific activity (units/mg)	Folds	Recovery (%)
Cell-free medium	1000	56.5	1648	29	1	100
40% and 80% (NH_4_)_2_SO_4_ precipitate	80	11	1145	104	4	69.5
SephadexG-200 gel-filtration	5	0.86	580	675	23	35.2
SephadexG-75 gel-filtration	2	0.235	285	1170	40	17.3

a Protein contents were determined by Bradford method;

b Carrageenase activity was determined by the reducing power method of 3,5-dinitrosalicylic acid (DNS).
